# Antimicrobial Resistance Across Four Iraqi Governorates: A Multicenter Cross-Sectional Surveillance Analysis With Implications for Antimicrobial Stewardship Policy and WHO AWaRe–Aligned Public Health Strategies

**DOI:** 10.2196/94436

**Published:** 2026-08-20

**Authors:** Hemn Omer, Omeed Darweesh, Gulbahar Karim, Baref Rashid, Rozhgar Ahmed, Andrew Seaton, Brian Godman, Amanj Kurdi

**Affiliations:** 1 College of Pharmacy, Al-Qalam University, Kirkuk, Kirkuk, Iraq; 2 College of Pharmacy, Al-Kitab University, Kirkuk 36015, Iraq; 3 Department of Basic Science, College of Nursing, University of Kirkuk, Kirkuk, Iraq; 4 Kurdistan Higher Council of Medical specialties, Erbil, Iraq; 5 Infection unit, Queen Elizabeth University Hospital, NHS Greater Glasgow and Clyde, Glasgow, Glasgow, United Kingdom; 6 Department of Public Health Pharmacy and Management, School of Pharmacy, Sefako Makgatho Health Sciences University, Pertoria, Gauteng, South Africa; 7 Strathclyde Institute of Pharmacy and Biomedical Sciences, University of Strathclyde, 161 Cathedral Street, Glasgow, Scotland, G4 0RE, United Kingdom, 44 07842434629; 8 Antibiotic Policy Group, Institute for Infection and Immunity, City St. George’s, University of London, , London, United Kingdom

**Keywords:** antimicrobial resistance, surveillance, multidrug resistance, WHO AWaRe, antimicrobial stewardship, public health policy, health systems strengthening., World Health Organization Access Watch Reserve

## Abstract

**Background:**

Antimicrobial resistance is a major public health threat globally, with disproportionate impact in low- and middle-income countries, where surveillance systems are often fragmented. In Iraq, nationally integrated multicenter resistance data to support stewardship policy and World Health Organization Access Watch Reserve (WHO AWaRe)–aligned interventions remain limited.

**Objective:**

The objective of this study is to characterize multicenter antimicrobial resistance patterns across inpatient and outpatient laboratory settings in selected Iraqi governorates, and evaluate their implications for surveillance strengthening and antimicrobial stewardship policy.

**Methods:**

We performed a multicenter cross-sectional surveillance analysis of routine microbiology data from four Iraqi governorates, representing approximately 10% of Iraq’s population. Deduplicated isolates (first isolate per patient per specimen type) were included. Multidrug resistance was defined as non-susceptibility to at least one agent in three or more antimicrobial classes. Resistance proportions with 95% CI were calculated and compared by clinical setting and specimen type.

**Results:**

Among 846 isolates, *Escherichia coli* (n=235/846, 27.8%), *Staphylococcus aureus* (n=151/846, 17.9%), *Pseudomonas aeruginosa* (n=65/846, 7.7%), and *Klebsiella pneumoniae* (n=59/846, 7.0%) predominated. Overall multidrug resistance prevalence was 57.3% (95% CI 53.9‐60.6) and was significantly higher among inpatients (*P*=.04). Third-generation cephalosporin resistance was detected in 69.3% (106/153) of *Escherichia coli* and 44.4% (8/18) of *Klebsiella pneumoniae *isolates. Carbapenem resistance was present in 12.4% (20/161) of *Escherichia coli*, 11.1% (2/18) of *Klebsiella pneumoniae*, and 29.4% (5/17) of *Pseudomonas aeruginosa*. Methicillin resistance was identified in 89.8% (136/151) of *Staphylococcus aureus *isolates.

**Conclusions:**

High multidrug resistance prevalence and substantial resistance among priority pathogens highlight urgent needs for strengthened surveillance integration, routine antibiogram reporting, and antimicrobial stewardship interventions aligned with WHO AWaRe targets. These findings provide multicenter subnational evidence to support surveillance strengthening, routine antibiogram reporting, and antimicrobial stewardship policy refinement in Iraq.

## Introduction

Antimicrobial resistance (AMR) is a major global public health challenge that threatens progress toward universal health coverage and sustainable development. According to the 2019 global burden of AMR analysis, bacterial AMR was estimated to be directly attributable to approximately 1.27 million deaths globally, with a further 4.95 million associated deaths, disproportionately affecting low- and middle-income countries (LMICs) where surveillance capacity is often limited [1-3]. In fragile and postconflict settings such as Iraq, structural challenges affecting laboratory capacity, antimicrobial regulation, and data integration may amplify resistance emergence and constrain effective surveillance and policy response [4,5].

Effective AMR response depends on robust, representative, and integrated surveillance systems capable of generating actionable data for clinicians, policymakers, and public health authorities. Surveillance data not only inform empiric treatment guidelines and antimicrobial stewardship (AMS) interventions, but also underpin national action planning, resource allocation, and monitoring of progress toward World Health Organization (WHO) and United Nations AMR targets. The WHO established the Global Antimicrobial Resistance and Use Surveillance System (GLASS) to standardize AMR reporting and strengthen national surveillance capacity [6]. However, while GLASS provides an essential global surveillance framework, important methodological and structural constraints affect interpretation of country-specific data, particularly in LMICs with incomplete laboratory coverage, nonstandardized testing panels, and limited denominator information [6]. In many participating countries, including Iraq, surveillance data are derived from selected sentinel laboratories without systematic linkage to population denominators, health care usage metrics, or standardized case definitions. Addressing these gaps requires complementary analyses integrating deduplicated, isolate-level data across multiple centers to improve operational relevance and local interpretability. In Iraq, available AMR data are largely derived from selected sentinel laboratories and may not fully capture national pathogen distribution, rural–urban variation, private-sector testing, or health care-associated infection dynamics [7,8]. This is important as the Middle East has been identified as a region with a high and growing burden of AMR, driven by a combination of health system fragility, regulatory challenges, and heterogeneous laboratory infrastructures [5]. Iraq represents a critical case study for AMR surveillance strengthening in fragile health systems. Decades of conflict, economic instability, and infrastructure disruption have affected laboratory networks, data integration systems, infection prevention capacity, and antimicrobial governance frameworks [9]. While Iraq participates in GLASS and has developed a national action plan on AMR (2018‐2022), implementation challenges have been documented, including fragmented surveillance systems, limited routine data integration, and variable enforcement of antimicrobial regulations [9,10]. This is similar to other LMICs that experience difficulties with meeting national action plan goals due to concerns with available resources as well as personnel [11],

Published studies from Iraq consistently report high levels of resistance among key bacterial pathogens. Multidrug-resistant (MDR) *Acinetobacter spp*., *extended-spectrum beta-lactamase* (ESBL)*-*producing *Escherichia coli,* and *Klebsiella pneumoniae*, *carbapenem-resistant Pseudomonas aeruginosa*, and *methicillin-resistant Staphylococcus aureus (MRSA*) have been described across multiple hospital settings [4,8,12-15]. Intensive care unit-based studies have further demonstrated discordance between empiric antibiotic prescribing and susceptibility results, reflecting both diagnostic constraints and evolving resistance profiles [4,7,16]. Community-derived isolates have also shown increasing resistance to commonly used antibiotics, raising concerns regarding first-line treatment effectiveness [4,9].

Despite this growing body of evidence, AMR data in Iraq remain fragmented across provinces, institutions, and study designs, with limited integration into a coordinated national surveillance platform. To date, no multicenter analysis has synthesized deduplicated hospital and community resistance data across major bacterial pathogens while explicitly contextualizing findings within GLASS reporting and WHO Access Watch Reserve (AWaRe) stewardship frameworks. Iraq occupies a strategic geographic position with substantial population mobility, cross-border health care usage, regional displacement, and international travel. Resistant organisms emerging in fragile or conflict-affected settings may disseminate regionally and globally through migration, cross-border health care usage, and population displacement, reinforcing the importance of strengthening surveillance systems beyond national boundaries [17]. In addition, patterns observed in Iraq reflect challenges common to many LMIC and postconflict health systems, including limited diagnostic infrastructure, empiric prescribing pressures, and uneven surveillance integration. Consequently, characterizing current AMR patterns in Iraq contributes to the global understanding of AMR epidemiology in fragile settings and provides transferable lessons for surveillance strengthening and empiric therapy optimization internationally. In settings where empiric therapy is frequently initiated prior to culture results, absence of consolidated national resistance data may contribute to suboptimal treatment selection and further resistance selection pressure [7,18,19]. This gap can be partly addressed by conducting a systematic national synthesis of AMR patterns in Iraq. Such an analysis can clarify pathogen-specific resistance distributions, identify priority resistance threats, contextualize Iraqi data within global estimates, and provide an evidence base to inform empiric treatment recommendations and surveillance strengthening. Consequently, this study aims to generate a multicenter, deduplicated synthesis of bacterial AMR patterns across inpatient and outpatient laboratory settings in selected Iraqi governorates in Iraq and to evaluate their implications for strengthening surveillance integration, stewardship policy reform, and WHO AWaRe–aligned public health strategies.

## Methods

### Study Design and Setting

This study was designed as a multicenter cross-sectional surveillance analysis of routinely collected microbiology laboratory data from four Iraqi governorates: Kirkuk, Tikrit, Sulaymaniyah, and Mosul. The primary objective was descriptive surveillance characterization of resistance patterns across participating laboratories rather than inferential epidemiological estimation or causal assessment of resistance determinants. Accordingly, the findings should be interpreted within the context of routine operational surveillance data generated through clinical diagnostic practice. These provinces were purposively selected to provide geographically and demographically diverse surveillance coverage; however, they were not intended to constitute a nationally representative sampling framework. Collectively, the included catchment areas cover approximately 10% of Iraq’s estimated population of 47 million. The inclusion of multiple provinces was intended to overcome the limitations of prior Iraqi studies [12,14,15,19]; which were often restricted to single cities, and/or pathogens. In each governorate, data were obtained from the principal tertiary public hospital laboratory, which functions as the primary referral microbiology center for surrounding districts. These laboratories constitute major public-sector diagnostic nodes within their respective regions, although they do not encompass private laboratories or smaller peripheral facilities. Data were collected between July and December 2025 and included isolates from both inpatient and outpatient settings. Outpatient status should not be interpreted as equivalent to community-acquired infection, as information on prior health care exposure, referral history, recent hospitalization, or previous antibiotic use was not available within the dataset. Microbiological testing was ordered according to routine clinician-driven practice rather than through a predefined surveillance sampling protocol. Testing is provided free of charge within participating public hospitals. As denominator data on total admissions, outpatient visits, and infection episodes were unavailable, findings represent laboratory-confirmed resistance patterns among tested patients rather than population incidence or prevalence estimates. Because testing followed routine clinician-directed practice rather than structured sampling, the dataset may preferentially capture clinically complex, severe, treatment-refractory, or diagnostically investigated cases. Consequently, the findings should be interpreted as operational surveillance observations, and the scope of inference is restricted to describing resistance distributions within participating laboratory settings rather than deriving nationally representative epidemiological estimates. This analysis therefore reflects operational surveillance data derived from routine diagnostic activity rather than structured sentinel sampling. The study was conducted and reported in accordance with the Strengthening the Reporting of Observational Studies in Epidemiology

guidance for cross-sectional observational studies [20]; (Checklist 1) and aligns with WHO GLASS methodological principles for isolate-based AMR surveillance [21].

### Study Population and Eligibility Criteria

All patients of any age or sex with at least one positive bacterial culture during the study period were eligible for inclusion. Extracted variables included patient age, sex, inpatient or outpatient status, governorate, specimen type, bacterial species identification, and antimicrobial susceptibility testing (AST) results. Anonymized laboratory records were extracted retrospectively from the electronic laboratory information systems of the principal tertiary public hospital laboratory in each participating governorate using a standardized data extraction template. Data extraction was undertaken locally by designated laboratory personnel, after which records were anonymized, quality checked for completeness and consistency, deduplicated in accordance with WHO GLASS guidance, and combined into a single dataset for analysis. To minimize duplication and align with international surveillance standards, only the first isolate per patient per specimen type during the study period was included in the analysis. Repeat isolates of the same organism from the same patient and specimen source were excluded, consistent with WHO GLASS deduplication guidance [21]. Negative cultures, fungal-only isolates, and records with incomplete susceptibility or missing key demographic variables were excluded. Unexpected or discrepant susceptibility profiles underwent internal laboratory review and repeat testing using disk diffusion and/or VITEK 2 (bioMérieux) confirmation where applicable. Isolates lacking essential susceptibility information required for resistance phenotype assignment, including MDR classification, were excluded from final analysis. Data were anonymized prior to aggregation and analysis to ensure compliance with ethical and data governance standards.

### Specimen Collection and Microbiological Identification

Specimens were obtained as part of routine clinical care in accordance with standard clinical microbiology practice. Specimen types included urine, blood, wound swabs, vaginal swabs, throat swabs, stool samples, respiratory tract specimens, and sterile body fluids such as cerebrospinal, pleural, synovial, and ascitic fluids. Blood cultures were processed using automated systems including BACT or ALERT 3D (bioMérieux) and BD BACTEC FX40 (Becton, Dickinson and Company). Other specimens were inoculated onto appropriate selective and differential media including blood agar, chocolate agar, and Brilliance UTI Clarity agar (Oxoid). Primary bacterial identification was performed locally using routine microbiological workflows combining conventional phenotypic and biochemical methods with automated platforms according to laboratory practice. Standard biochemical testing included Gram staining, catalase, and coagulase testing for Gram-positive cocci, and oxidase, triple sugar iron, citrate, and related biochemical assays for Gram-negative bacilli. The VITEK 2 Compact automated system was used for automated organism identification and susceptibility confirmation, particularly for selected MDR, complex, or diagnostically challenging isolates including *P. aeruginosa* and *K. pneumonia*. Matrix-assisted laser desorption/ionization time-of-flight mass spectrometry (MALDI-TOF MS) [22]; was not available across all participating centers but was used centrally at the Infectious Diseases Control Center in Baghdad as a confirmatory reference method for isolates with atypical biochemical profiles, unusual clinical contexts, or suspected novel resistance phenotypes.

### AST

AST was performed using Kirby–Bauer disk diffusion, VITEK 2 automated testing, and/or minimum inhibitory concentration (MIC)-based methods depending on organism type, isolate complexity, and institutional laboratory protocol. Kirby–Bauer disk diffusion represented the primary AST method across participating laboratories, while VITEK 2 and MIC-based approaches were used for selected isolates requiring confirmatory susceptibility testing, particularly suspected MDR organisms and selected *P. aeruginosa* and *K. pneumonia* isolates. AST interpretation was standardized across centers using Clinical and Laboratory Standards Institute (CLSI) M100, 35th edition (2025) criteria [23]. All susceptibility categories and MIC breakpoints were interpreted according to the 2025 standards to support intercenter comparability. Although routine antibiotic panels varied modestly across centers because of organism-specific testing practices, reagent availability, and local laboratory capacity, laboratories maintained core pathogen-specific antibiotic panels to support consistent classification of multidrug resistance. Routine quality control procedures were implemented using standard reference strains, including *E. coli* American Type Culture Collection 25922 and *S. aureus* American Type Culture Collection 25923, tested monthly and when new reagent or disk batches were introduced, in accordance with CLSI M100-2025 guidance. For MRSA classification, cefoxitin disk diffusion (30 µg) was used as the primary standardized screening method across participating centers in accordance with CLSI recommendations. Although oxacillin was used in a limited number of local workflows, all isolates included in the final MRSA analysis were validated using cefoxitin criteria to ensure uniform classification. Carbapenem resistance was defined as nonsusceptibility to at least one tested carbapenem agent (imipenem, meropenem, and/or ertapenem) according to CLSI M100-2025 criteria. To mitigate interlaboratory heterogeneity, interpretive standardization was maintained through uniform application of CLSI M100-2025 criteria across all testing platforms. Discrepant or unusual resistance phenotypes underwent confirmatory assessment through advanced methods at the Infectious Diseases Control Center reference laboratory where required. Glycopeptide susceptibility findings, including vancomycin non-susceptibility among S. aureus, should be interpreted within the context of routine multicenter laboratory surveillance data, recognizing that confirmatory MIC testing and reference laboratory validation were not uniformly available for all isolates. These standards provide internationally accepted breakpoints for categorizing isolates as susceptible, intermediate, or resistant. For surveillance consistency and conservative public health estimation, isolates categorized as intermediate were grouped with resistant isolates, consistent with WHO GLASS analytical methodology [21]. MDR was defined as non-susceptibility to at least one agent in three or more antimicrobial classes, in accordance with the internationally standardized definitions proposed by Magiorakos et al [24]. Terminology throughout the manuscript was aligned with WHO GLASS reporting conventions. The term “non-susceptible” was used for AST outcomes where appropriate, while specific resistance terminology was retained for recognized resistance phenotypes such as MRSA, carbapenem-resistant organisms, and intrinsic resistance classifications.

### Statistical Analysis

Descriptive surveillance analytics were applied to summarize demographic characteristics, specimen distribution, pathogen prevalence, and AMR proportions. Given the surveillance-oriented design, analyses were intentionally descriptive and exploratory, aiming to characterize operational resistance patterns across clinical settings and specimen types rather than test causal hypotheses or estimate independent risk relationships. Survey weighting was not applied because the dataset comprised all eligible deduplicated isolates obtained through routine laboratory surveillance from participating centers rather than a probability-based sample of the Iraqi population. Consequently, no sampling probabilities or survey weights were available or appropriate, and all analyses were performed using unweighted data. Although selected demographic and laboratory variables were available, the dataset lacked several key determinants required for robust adjusted modeling, including prior antibiotic exposure, recent hospitalization, health care contact history, comorbidity burden, invasive procedures, intensive care unit admission, infection severity, and prior microbiological history. Consequently, multivariable modeling was not undertaken because adjusted estimates based on incompletely measured resistance determinants could be vulnerable to substantial residual confounding and potentially misleading causal interpretation. Resistance rates were calculated as the proportion of resistant isolates among tested isolates for each pathogen–antibiotic combination, consistent with WHO GLASS reporting methodology [21]. Proportions were reported with 95% CI to quantify statistical uncertainty. Comparisons between inpatient and outpatient populations, across governorates, and between specimen types were conducted using χ^2^ or Fisher exact tests as appropriate. These subgroup comparisons were intended as descriptive exploratory analyses to characterize variation across clinical contexts rather than to identify independent predictors of resistance. Accordingly, multivariable modeling and formal adjustment for multiple comparisons were not undertaken because they were outside the primary surveillance objectives of this study. Records with missing key demographic variables or incomplete susceptibility information required for resistance phenotype classification were excluded during data cleaning, as described in the eligibility criteria. Analyses were therefore performed using complete available data for each relevant pathogen–antibiotic combination, resulting in variable denominators across selected comparisons. A two-sided *P* value <.05 was considered statistically significant. Statistical analyses were performed using SPSS version 28.0 (IBM Corp).

### Ethical Considerations

Ethical approval was obtained from the Institutional Review Board of Al-Kitab University (approval number: 6/557). As the study involved retrospective analysis of anonymized laboratory data without direct patient contact, the requirement for informed consent was waived. All procedures were conducted in accordance with the Declaration of Helsinki and applicable national ethical standards [25]. Data were handled in accordance with institutional data governance procedures, and no identifiable patient information was retained within the analytical dataset.

## Results

### Study Population and Specimen Characteristics

A total of 846 deduplicated bacterial isolates were included in this multicenter surveillance dataset. Urine specimens accounted for over half of isolates (n=449, 53.0%), followed by vaginal swabs (119/846, 14.0%), wound swabs (95/846, 11.2%), and blood cultures (86/846, 10.1%) (Table 1). The predominance of urine specimens is consistent with routine diagnostic testing patterns and indicates that urinary tract infections constitute a major driver of microbiological sampling within participating centers. Two-thirds of isolates were obtained from female patients (559/846, 66.0%), consistent with the high proportion of urinary and vaginal specimens. The largest age group represented was 21‐30 years (194/846, 22.9%). Most isolates were derived from outpatient settings (535/846, 63.2%), indicating that a substantial proportion of detected resistance was identified among isolates obtained through outpatient clinical pathways. However, outpatient status does not necessarily imply community-acquired infection because health care exposure history was unavailable. Geographically, isolates were distributed across four governorates, with Kirkuk contributing 43.9% (372/846) and Tikrit 25.1% (213/846), reflecting laboratory catchment activity rather than population-weighted sampling.

**Table 1.** Demographic and clinical characteristics of unique patients (N=846) and specimen distribution of bacterial isolates across four Iraqi governorates^a^.

Variables and categories		Total, N (%)	Urine, n (%)	Vaginal swab, n (%)	Wound swab n (%)	Blood, n (%)	Throat swab, n (%)	Stool, n (%)
Total								
Total patients		846 (100)	449 (53.0)	119 (14.0)	95 (11.2)	86 (10.1)	68 (8.0)	29 (3.4)
Sex								
Male		287 (33.9)	154 (53.6)	0 (0)	51 (17.7)	43 (14.9)	27 (9.4)	12 (4.1)
Female		559 (66.1)	295 (52.7)	119 (21.2)	44 (7.8)	43 (7.6)	41 (7.3)	17 (3.0)
Age, years								
0‐2		36 (4.2)	16 (44.4)	0 (0)	6 (16.6)	10 (27.7)	3 (8.3)	1 (2.7)
>2‐12		57 (6.7)	31 (54.3)	0 (0)	7 (12.2)	13 (22.8)	1 (1.7)	5 (8.7)
13‐20		103 (12.1)	47 (45.6)	11 (10.6)	14 (13.5)	8 (7.7)	17 (16.5)	6 (5.8)
21‐30		194 (22.9)	109 (56.1)	34 (17.5)	22 (11.3)	18 (9.2)	6 (3.0)	5 (2.5)
31‐40		173 (20.4)	87 (50.2)	38 (21.9)	18 (10.4)	10 (5.7)	14 (8.0)	6 (3.4)
41‐50		98 (11.5)	58 (59.1)	19 (19.3)	8 (8.1)	6 (6.1)	5 (5.1)	2 (2.0)
51‐60		68 (8.0)	30 (44.1)	10 (14.7)	5 (7.3)	8 (11.7)	14 (20.6)	1 (1.4)
>60		117 (13.8)	71 (60.7)	7 (5.9)	15 (12.8)	13 (11.1)	8 (6.8)	3 (2.5)
Patient status								
Outpatient		535 (63.2)	307 (57.3)	94 (17.5)	56 (10.4)	13 (2.4)	48 (8.9)	17 (3.1)
Inpatient		311 (36.7)	142 (45.6)	25 (8.0)	39 (12.5)	73 (23.4)	20 (6.4)	12 (3.8)
Governorate distribution								
Kirkuk		372 (43.9)	196 (52.7)	32 (8.6)	37 (9.9)	70 (18.8)	22 (5.9)	15 (4.0)
Tikrit		213 (25.1)	115 (53.9)	54 (25.3)	15 (7.0)	0 (0)	19 (8.9)	10 (4.6)
Sulaymaniyah		138 (16.3)	63 (45.6)	25 (18.1)	19 (13.7)	14 (10.1)	17 (12.3)	0 (0.0)
Mosul		123 (14.5)	75 (60.9)	8 (6.5)	24 (19.5)	2 (1.6)	10 (8.1)	4 (3.2)

a Percentages in the total column are calculated relative to all included patients. Percentages presented across specimen columns are row percentages showing the distribution of each demographic or clinical subgroup across specimen categories; therefore, percentages within rows may sum to approximately 100% owing to rounding.

### Distribution of Bacterial Pathogens

**Figure 1.**
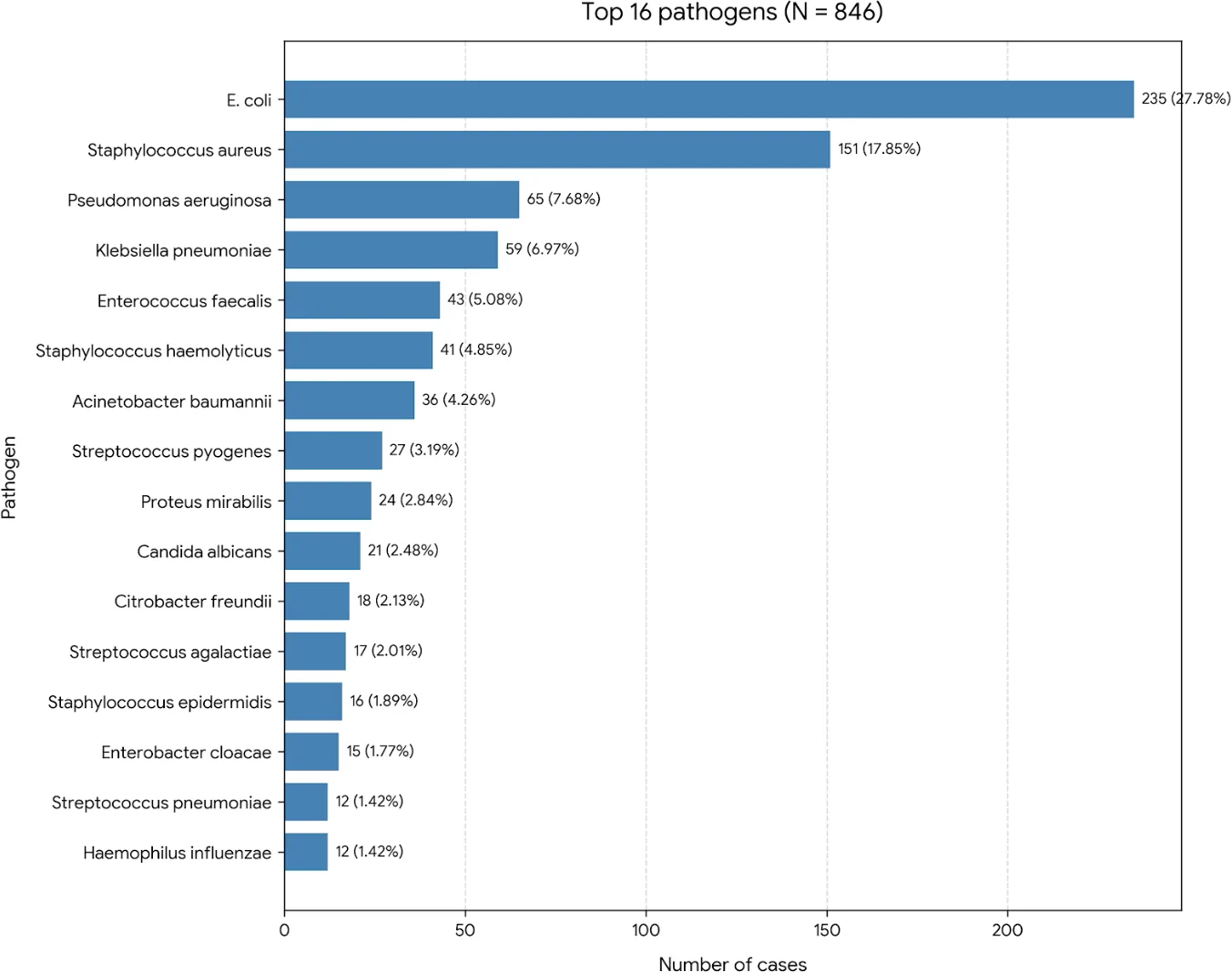
Distribution of the top 16 bacterial pathogens isolated from clinical specimens in four Iraqi Governorates (N=846).

**Figure 2.**
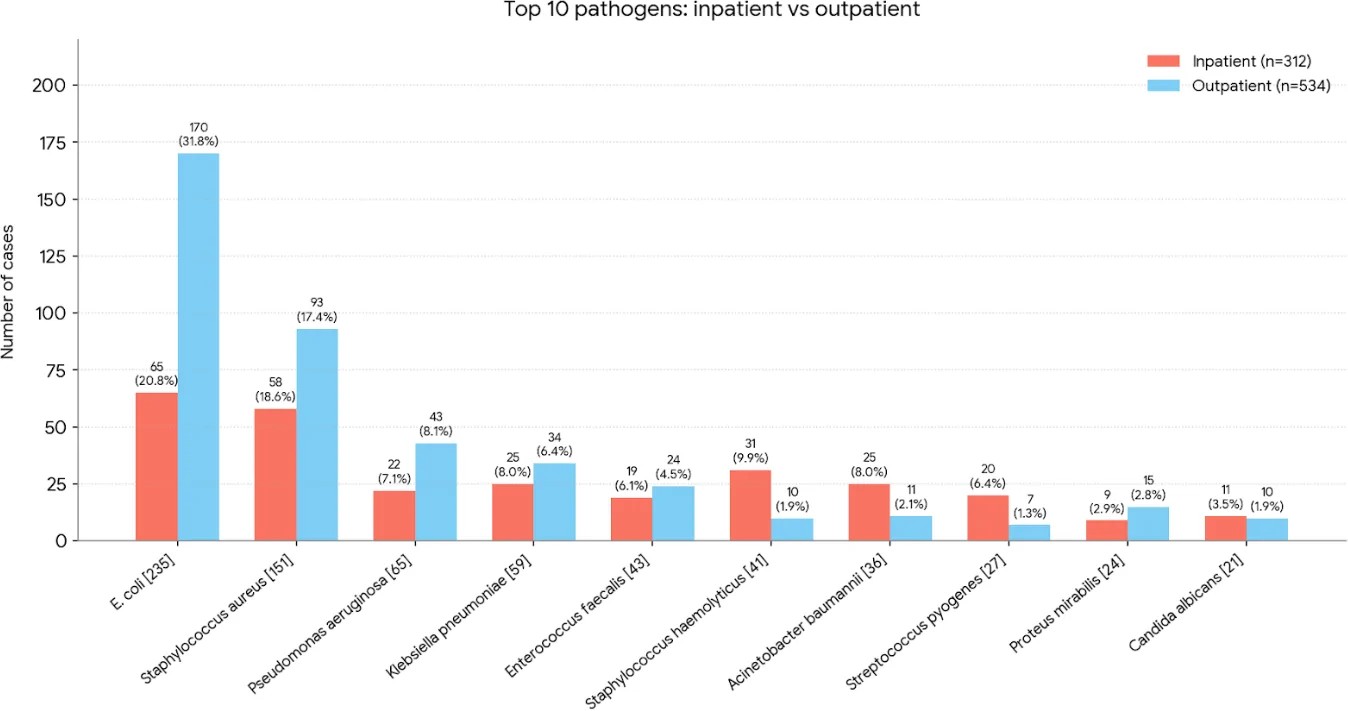
Distribution of the top 10 bacterial pathogens by clinical setting (Inpatient vs outpatient) in four Iraqi Governorates.

Among the 846 isolates, more than 20 bacterial species were identified. *E. coli* was the most frequently isolated pathogen (n=235, 27.78%), followed by *S. aureus* (n=151, 17.85%), *P. aeruginosa* (n=65, 7.68%), and *K. pneumonia* (n=59, 6.97%). Together, these four pathogens accounted for more than 60% (510/846) of all isolates. Other notable pathogens included *Enterococcus faecalis* (n/N43/846, 5.08%), *Staphylococcus haemolyticus* (41/846, 4.85%), *Acinetobacter baumannii* (36/846, 4.26%), and *Streptococcus pyogenes* (27/846, 3.19%) (Figure 1). The distribution of the top 16 most common pathogens demonstrates the predominance of Gram-negative organisms, particularly *E. coli*. Comparison between inpatient and outpatient populations showed variation in pathogen distribution (Figure 2). *E. coli* was more frequently identified among outpatients, whereas *Acinetobacter baumannii* and *Staphylococcus haemolyticus* were proportionally more common among inpatients.

### Composite AMR Indicators and MDR

**Figure 3.**
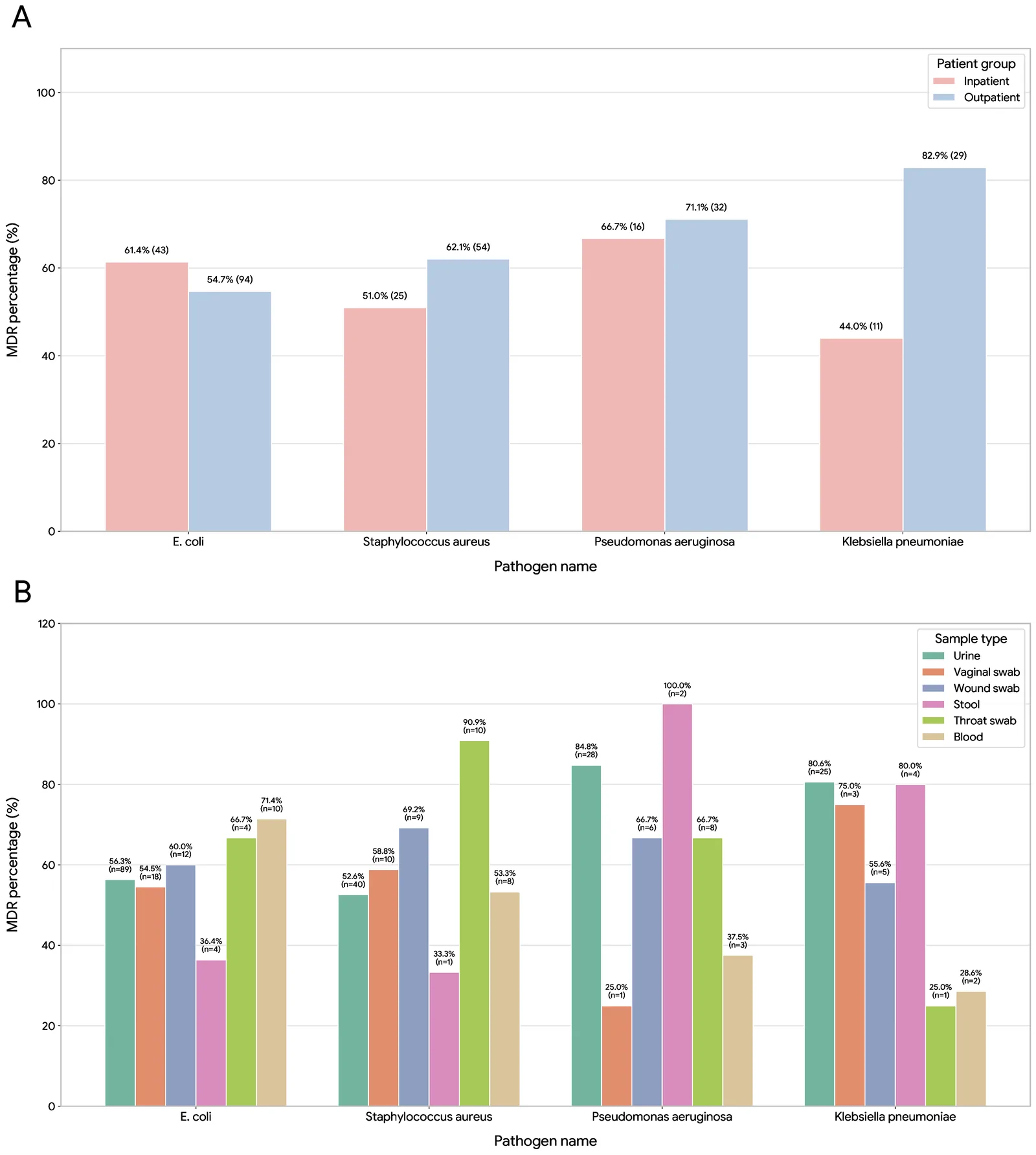
Multidrug resistance prevalence by (A) clinical setting and (B) specimen type among major bacterial pathogens in Iraq. MDR was defined as resistance to at least one antimicrobial agent in three or more antimicrobial classes. MDR: multidrug resistant;

The overall prevalence of MDR, defined as resistance to at least one agent in three or more antimicrobial classes, was 57.3% (485/846, 95% CI 53.9‐60.6). MDR was significantly more frequent among inpatients than outpatients (62.7%, 195/311, 95% CI 57.2‐68.1 vs 54.2% 290/535, 95% CI 49.9‐58.4; *P*=.04), which is consistent with a greater concentration of resistant organisms within hospital settings. However, these comparisons reflect descriptive variation across clinical settings and should not be interpreted as evidence of independent associations because adjustment for important patient- and health care-level confounders was not feasible within the available surveillance dataset. When analyzed at the pathogen level, however, differences in MDR prevalence between inpatient and outpatient isolates were not statistically significant for *E. coli* (*P*=.39), *S. aureus* (*P*=.28), or *P. aeruginosa* (*P*=.79). In contrast, MDR prevalence differed significantly for *K. pneumonia* between clinical settings (*P*=.002, Fisher exact test), suggesting pathogen-specific variation in health care-associated resistance dynamics (Figure 3A).

Among the most common pathogens, MDR prevalence was highest in *P. aeruginosa* (71.6%, 47/65, 95% CI 59.6‐81.2), followed by *K. pneumonia* (66.1%, 39/59, 95% CI 53.3‐76.8), *S. aureus* (58.3%, 88/151, 95% CI 50.3‐65.9), and *E. coli* (56.6%, 133/235, 95% CI 50.2‐62.8). Carbapenem resistance, defined as resistance to at least one carbapenem (imipenem and/or meropenem) among isolates tested for carbapenems, was observed in 12.4% of *E. coli* (20/161, 95% CI 8.2‐18.3), 11.1% of *K. pneumoniae* (2/18, 95% CI 3.1‐32.8), and 29.4% of *P. aeruginosa* (5/17, 95% CI 13.3‐53.1). Although denominators varied according to testing availability, carbapenem resistance was consistently present across key Gram-negative pathogens. Third-generation cephalosporin resistance, used as a surveillance proxy indicator for probable ESBL activity rather than confirmed ESBL production and defined as resistance to at least one of ceftriaxone, ceftazidime, or cefotaxime among isolates tested for these agents, was detected in 69.3% of *E. coli* (106/153, 95% CI 61.6‐76.0) and 44.4% of *K. pneumoniae* (8/18, 95% CI 24.6‐66.3). At the genus level, 70.0% of *Klebsiella* spp. isolates tested (21/30, 95% CI 52.1‐83.3) demonstrated third-generation cephalosporin resistance. MRSA, defined by oxacillin resistance, accounted for 89.8% of *S. aureus* isolates (136/151, 95% CI 83.9‐93.6), reflecting a markedly elevated burden of methicillin resistance.

### Specimen-Specific Multidrug Resistance (MDR) Variation

Analysis of MDR prevalence by specimen type demonstrated significant variation for selected pathogens. Among *K. pneumonia*, urinary isolates exhibited substantially higher MDR prevalence compared with blood isolates (80.6% vs 28.6%; *P*=.01) and throat swab isolates (80.6% vs 25.0%; *P*=.04). Similarly, *P. aeruginosa* showed significantly higher MDR prevalence in urine compared with blood (84.8% vs 37.5%; *P*=.01) and vaginal swabs (84.8% vs 25.0%; *P*=.02). For *S. aureus*, throat swab isolates demonstrated significantly higher MDR prevalence than urinary isolates (90.9% vs 52.6%; *P*=.02). No statistically significant specimen-level differences were observed for *E. coli* (all *P*>.05) (Figure 3B). These findings indicate that resistance burden varies not only by pathogen and clinical setting but also by anatomical source, with urinary isolates of certain Gram-negative pathogens demonstrating particularly elevated MDR prevalence.

### AMR Patterns Among Major Gram-Negative Pathogens

#### E. Coli

**Figure 4.**
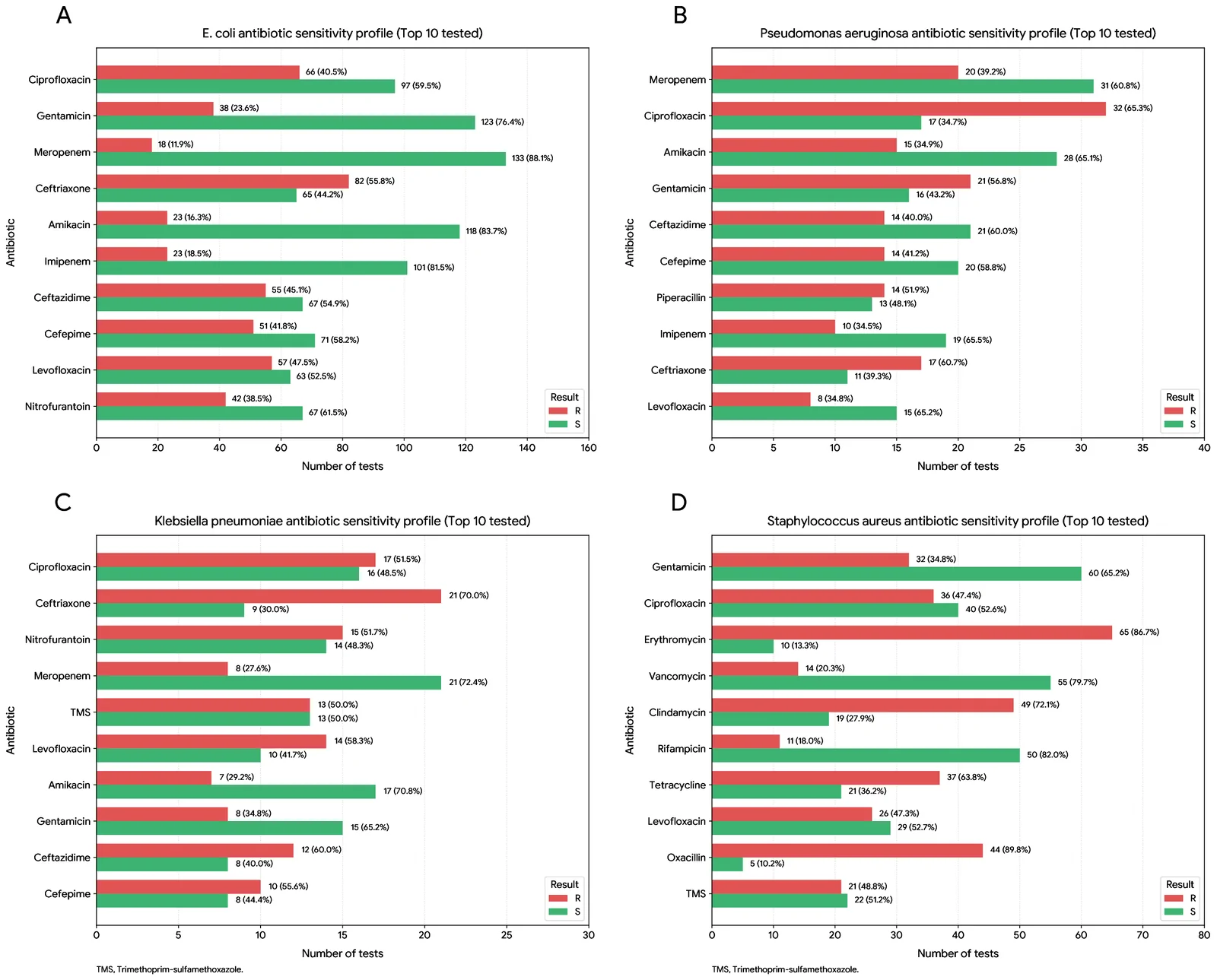
Antimicrobial susceptibility profiles of major bacterial pathogens isolated in four Iraqi governorates. (A) Escherichia coli; (B) *Pseudomonas aeruginosa*; (C) Klebsiella pneumoniae*; (*D*)* Staphylococcus aureus. Bars represent proportions of resistant (R) and susceptible (S) isolates among tested samples for each pathogen–antibiotic combination. Denominators varied according to organism-specific testing panels, antibiotic availability, and laboratory testing practices across participating centers. R: resistant; S: susceptible.

Resistance rates among *E. coli* isolates varied substantially across antibiotic classes (Figure 4A). High resistance was observed to ceftriaxone (82/147, 55.8%), ceftazidime (55/122, 45.1%), and ciprofloxacin (66/163, 40.5%). Resistance to levofloxacin was 47.5% (57/120), and to cefepime 41.8% (51/122). Nitrofurantoin resistance was 38.5% (42/109). Lower resistance rates were observed for carbapenems and aminoglycosides. Resistance to meropenem was 11.9% (18/151), and to imipenem 18.5% (23/124). Amikacin resistance was 16.3% (23/141), and gentamicin resistance 23.6% (38/161).

#### P. Aeruginosa

Among *P. aeruginosa* isolates, resistance to ciprofloxacin was 65.3% (32/49), representing the highest resistance observed in this species (Figure 4B). Gentamicin resistance was 56.8% (21/37), and piperacillin resistance 51.9% (14/27). Ceftriaxone nonsusceptibility was observed in 60.7% of isolates; however, because *P. aeruginosa* is intrinsically nonsusceptible to ceftriaxone, this finding is not clinically informative as a marker of acquired resistance or therapeutic option loss. Carbapenem resistance was notable, with 39.2% (20/51) resistance to meropenem and 34.5% (10/29) to imipenem. Amikacin resistance was 34.9% (15/36). Resistance to ceftazidime and cefepime was 40.0% (14/35) and 41.2% (14/34), respectively (Figure 4B).

#### K. Pneumoniae

*K. pneumoniae* demonstrated high resistance to ceftriaxone (21/30, 70.0%) and ceftazidime (12/20, 60.0%). Resistance to ciprofloxacin was 51.5% (17/33), and to levofloxacin 58.3% (14/24). Nitrofurantoin resistance was 51.7% (15/29). Carbapenem resistance was lower compared with third-generation cephalosporins, with 27.6% (8/29) resistance to meropenem. Aminoglycoside resistance was 29.2% (7/24) for amikacin and 34.8% (8/23) for gentamicin (Figure 3C).

#### AMR Patterns Among *S. Aureus*

Among *S. aureus* isolates, oxacillin resistance was 89.8% (44/49), consistent with a high prevalence of methicillin resistance. Erythromycin resistance was 86.7% (65/75), and clindamycin resistance 72.1% (49/68). Tetracycline resistance was 63.8% (37/58). Resistance to ciprofloxacin and levofloxacin was 47.4% (36/76) and 47.3% (26/55), respectively. Trimethoprim–sulfamethoxazole resistance was 48.8% (21/43). Gentamicin resistance was 34.8% (32/92) (Figure 4D). Vancomycin non-susceptibility was observed in 20.3% (14/69) of tested *S. aureus* isolates; however, this finding should be interpreted with considerable caution given the global rarity of confirmed vancomycin-resistant *S. aureus* (VRSA). Possible contributing explanations include testing methodology variability, misclassification of intermediate isolates as resistant, instrument- or platform-related limitations, lack of uniform confirmatory MIC testing, or potential data recording inconsistencies

## Discussion

### Key Findings

This multicenter surveillance analysis addressed the study objective of characterizing AMR patterns across hospital and outpatient laboratory settings in Iraq and evaluating their implications for surveillance strengthening and AMS policy. The findings demonstrated a high overall prevalence of multidrug resistance (485/846, 57.3%), substantial third-generation cephalosporin resistance among Enterobacterales, elevated carbapenem resistance among nonfermenters, and high methicillin resistance among *S. aureus*. The elevated third-generation cephalosporin resistance observed among Enterobacterales is consistent with a substantial burden of ESBL-probable organisms; however, these findings should be interpreted cautiously because phenotypic confirmatory testing and molecular characterization were not performed. Consequently, the results reflect surveillance indicators compatible with probable ESBL activity rather than microbiologically confirmed ESBL prevalence. Beyond the microbiological burden, these results underscore the urgent need for strengthened surveillance coordination, routine antibiogram reporting, and improved integration of laboratory data within broader AMR monitoring systems aligned with WHO GLASS and AWaRe frameworks. MDR prevalence was higher among inpatient isolates (195/311, 62.7%) than outpatient isolates (290/535, 54.2%), although resistance burden remained substantial across both health care settings, with resistant organisms observed across both inpatient and outpatient laboratory populations. However, outpatient isolates should not be interpreted as synonymous with community-acquired infection because prior health care exposure could not be assessed. Although MDR prevalence was higher among inpatients, resistance remained substantial across settings. Interpretation of subgroup differences warrants caution. Although higher MDR prevalence was observed among inpatient isolates and selected specimen categories, the available surveillance dataset did not include several important resistance determinants required for robust adjusted modeling, including previous antibiotic exposure, recent hospitalization, health care contact history, comorbidity burden, invasive procedures, intensive care unit admission, infection severity, or prior microbiological findings. Consequently, observed differences may partly reflect unmeasured variation in patient complexity, clinical indication, health care exposure, or testing practices rather than independent effects of clinical setting or specimen source alone. The present analysis therefore prioritized descriptive surveillance characterization over inferential modeling, consistent with the study’s operational surveillance objectives. These findings should be interpreted within Iraq’s broader AMR and surveillance landscape. Institute for Health Metrics and Evaluation (IHME) modeling estimated approximately 3450 AMR-attributable deaths in Iraq in 2021, with carbapenem-resistant Gram-negative organisms and MRSA contributing substantially to mortality. While such estimates rely on aggregated datasets, this study provides complementary operational laboratory data with context-specific resistance proportions relevant to stewardship planning and health system response. The observed high MRSA prevalence and substantial carbapenem resistance among *P. aeruginosa* and Enterobacterales are consistent with national mortality patterns. Recent Iraqi surveillance studies similarly report sustained high ESBL prevalence, increasing carbapenem resistance, and substantial resistance to third-generation cephalosporins and fluoroquinolones across multiple governorates and health care settings [8,18]. Regarding ceftriaxone findings in *P. aeruginosa*, it is important to note that *P. aeruginosa* is intrinsically nonsusceptible to most third-generation cephalosporins, including ceftriaxone, due to low outer membrane permeability, chromosomal AmpC β-lactamase production, and efflux mechanisms [26]. Accordingly, the ceftriaxone result is presented for completeness of the routine laboratory dataset but should not be interpreted as a clinically meaningful surveillance marker of acquired resistance or loss of a recommended therapeutic option, since ceftriaxone is not routinely indicated for *P. aeruginosa* infections [27].

### Interpretations, Implications, and Comparisons of the Study Findings in the Context of Existing Literature

#### Comparison With Global and Regional Surveillance

The WHO GLASS 2025 report includes Iraqi data from sentinel laboratories and reports resistance patterns broadly consistent with our findings. However, GLASS outputs are aggregated and adjusted for surveillance coverage, whereas this study provides deduplicated, center-level isolate data with inpatient–outpatient and specimen-level stratification, allowing greater insight into within-country and clinical-context variation [6]. Thus, this multicenter analysis complements rather than duplicates national reporting by enhancing granularity and operational relevance for stewardship and guideline refinement. Gram-negative pathogens dominated the resistance landscape, consistent with global and regional evidence identifying Enterobacterales and nonfermenters as priority AMR threats, particularly in Middle Eastern, postconflict, and resource-constrained settings [1,5,6]. Fragmented laboratory systems and inconsistent antimicrobial regulation, recognized drivers of AMR expansion in Iraq and neighboring countries, may contribute to these patterns [5]. The high prevalence of third-generation cephalosporin resistance in *E. coli* (106/153, 69.3%) and *K. pneumoniae* (8/18, 44.4%‐21/30, 70.0%) is particularly concerning and suggests a substantial burden of ESBL-probable Enterobacterales. However, because phenotypic ESBL confirmation and molecular testing were not undertaken, these observations should be interpreted as surveillance indicators of probable resistance mechanisms rather than confirmed ESBL prevalence estimates. GLASS 2025 reports persistent and, in some regions, rising resistance to third-generation cephalosporins among bloodstream and urinary isolates of *E. coli* and *K. pneumoniae*, especially in LMIC settings [6]. Our findings place Iraq toward the upper range of these global estimates and, importantly, demonstrate statistically significant inpatient–outpatient differences for *K. pneumoniae* and specimen-level heterogeneity for selected pathogens. Such localized variation has direct implications for empiric therapy optimization and AMS prioritization but cannot be discerned from national-level GLASS outputs alone. Rather than duplicating GLASS, this multicenter analysis complements national surveillance by providing operationally relevant, context-specific insights into resistance distribution across health care settings within Iraq.

Moreover, carbapenem resistance, although lower than cephalosporin resistance, was consistently present, particularly in *P. aeruginosa* (5/17, 29.4%). Carbapenem-resistant nonfermenters are recognized by WHO as critical priority pathogens [28], and even moderate resistance levels can restrict empiric therapy and increase mortality risk in severe infection [1,29]. The very high MRSA prevalence (136/151, 89.8%) exceeds many international benchmarks; rates above 50% are commonly considered markers of entrenched health care-associated transmission and IPC challenges [6]. Despite the overall resistance burden, some susceptibility patterns suggest limited stewardship opportunities. Among Gram-negative pathogens, amikacin showed lower resistance than third-generation cephalosporins and fluoroquinolones, indicating potential utility within empiric combination regimens for suspected MDR Gram-negative infections, pending susceptibility results. Among *S. aureus*, comparatively lower resistance to trimethoprim–sulfamethoxazole and rifampicin suggests that selected oral agents may retain utility in some culture-guided community or step-down treatment settings. Nevertheless, the high MRSA burden substantially limits empiric reliance on beta-lactams for suspected staphylococcal infections. Overall, these findings emphasize the importance of timely microbiological diagnostics to optimize therapy and minimize unnecessary escalation to watch or reserve antibiotics within the WHO AWaRe framework.

In addition, the observed vancomycin nonsusceptibility rate among *S. aureus* isolates warrants careful interpretation within a surveillance quality assurance context. While confirmed VRSA remains rare globally [6,30], a reported nonsusceptibility proportion of this magnitude would be expected to attract substantial microbiological scrutiny. Several methodological explanations should therefore be considered, including testing methodology variability, breakpoint interpretation differences, misclassification of intermediate isolates as resistant, instrument-related limitations, heterogeneous laboratory workflows, lack of universal confirmatory MIC testing, and possible data entry or transcription inconsistencies. Elevated phenotypic resistance rates have nevertheless been reported in several LMIC settings [27], often reflecting laboratory methodology limitations, heterogeneous vancomycin-intermediate *Staphylococcus aureus* (VISA) phenotypes, or high glycopeptide selection pressure rather than fully confirmed vancomycin-resistant strains. If true VRSA were present at this magnitude, the implications would be substantial, including restriction of last-line glycopeptide therapy, increased reliance on linezolid or daptomycin, and urgent reinforcement of infection prevention and control measures [31]. Consequently, these findings should not be interpreted as definitive evidence of confirmed VRSA prevalence but rather as a signal warranting confirmatory MIC-based testing, strengthened microbiological quality assurance, and further prospective surveillance investigation. Similar variability in phenotypic resistance reporting has been observed in recent Iraqi surveillance reports [32].

#### Implications for Iraqi National Antimicrobial Guidelines (INAG)

The Iraqi National Antimicrobial Guidelines (INAG) emphasize rational prescribing, culture-guided therapy, short treatment duration, and avoidance of unnecessary broad-spectrum antibiotics [33]. However, effective guideline implementation depends on timely, representative resistance data. The resistance patterns identified in this surveillance analysis suggest that periodic revision of INAG should be explicitly linked to structured national antibiogram reporting cycles. Embedding surveillance–guideline feedback mechanisms within policy frameworks would enhance responsiveness to evolving resistance patterns. The guidelines explicitly recommend using narrow-spectrum agents whenever effective and caution against broad-spectrum escalation unless clinically justified [33]. There are similar sentiments in the WHO AWaRe guidance following concerns with the robustness of national antibiotic guidelines from many LMICs [34,35]. However, the resistance patterns observed in this study have several potentially actionable implications for empiric prescribing policy and periodic INAG revision. High third-generation cephalosporin resistance among Enterobacterales may reduce the reliability of ceftriaxone-based empiric regimens, particularly for severe urinary tract, bloodstream, and intraabdominal infections where *E. coli* and *K. pneumoniae* are common pathogens. Elevated fluoroquinolone resistance may similarly limit commonly used oral step-down and outpatient treatment strategies. Conversely, comparatively lower resistance to amikacin across major Gram-negative pathogens suggests a possible role within carefully supervised empiric combination approaches for suspected MDR Gram-negative infections, pending culture and susceptibility results. The substantial MRSA burden further supports review of empiric antistaphylococcal coverage strategies in selected hospital-associated, surgical, and skin or soft tissue infection contexts. The INAG emphasizes that empiric therapy should be informed by local antibiogram data. These findings support a more formal surveillance-to-guideline feedback mechanism whereby routine regional antibiograms directly inform scheduled INAG revision cycles, syndrome-specific empiric recommendations, AWaRe stewardship priorities, and structured 48‐72-hour antimicrobial review and de-escalation processes. Our findings underscore the urgency of updating national and institutional antibiograms regularly and integrating resistance surveillance into guideline revision cycles, which is currently not happening despite growing AMR rates. Without periodic updates reflecting current resistance patterns, empiric recommendations risk being misaligned with microbiological reality. Importantly, INAG also advises avoiding unnecessary macrolides, tetracyclines, quinolones, and aminoglycosides in pregnancy and pediatric populations. Rising resistance to these agents may further complicate safe empiric therapy selection in vulnerable groups.

### Alignment With WHO AWaRe Framework

The WHO AWaRe classification categorizes antibiotics into access, watch, and reserve groups to optimize stewardship and preserve last-line agents, which typically include watch and reserve antibiotics [36,37]. The WHO recommended that at least 60% of national antibiotic consumption should derive from the access group to help limit the use of watch and reserve antibiotics, particularly in LMICs, where the use of broader-spectrum agents may contribute to increased resistance selection pressure [37]. The United Nations General Assembly (UN-GA) recently increased this to 70% access antibiotics across sectors to reduce AMR [38,39]. In this study, resistance levels were particularly high for several watch-group antibiotics, including fluoroquinolones and third-generation cephalosporins, patterns consistent with selective pressure reported across LMICs [29,39,40]. Carbapenems remain critical for treating ESBL-producing Enterobacterales, and the presence of carbapenem resistance signals potential erosion of these last-line therapies. WHO AWaRe guidance emphasizes restricting watch and reserve antibiotics to appropriate indications and reinforcing microbiological testing before escalation [41]. Our findings therefore reinforce three key AWaRe-aligned priorities for Iraq, including strengthening Access antibiotic reliability through surveillance, reducing unnecessary watch antibiotic use, expanding AWaRe-based usage monitoring to inform AMS activities [42], and preserving carbapenems through formal stewardship and preauthorization systems, particularly given concerns regarding hospital antibiotic prescribing in Iraq [43].

The higher MDR prevalence among inpatients compared with outpatients is consistent with the recognized association between health care exposure to antibiotics and resistant infection risk reported in previous literature [29]. However, the descriptive design of this study does not permit causal attribution or assessment of independent risk factors. Hospital environments concentrate antibiotic use, invasive procedures, and vulnerable patients, all of which amplify resistance selection. However, the presence of substantial resistance among outpatient isolates demonstrates that resistant organisms were identified beyond inpatient settings, with an increasing focus on community antibiotic use in recent years given their appreciable prescribing in this sector versus hospital sectors [44,45]; however, outpatient isolates may include patients with previous health care contact, referral pathways, or other unmeasured health care exposures. Concerns with community dispensing of antibiotics in Iraq are not helped by high rates of their dispensing without a prescription [7,45-47], which may contribute to antimicrobial selection pressure. In addition, concerns with current nurse prescribing in ambulatory care clinics [46]. Both situations are not helped by concerns with current patient knowledge regarding antibiotics, AMR, and AMS in Iraq [45]. Consequently, addressing AMR in Iraq therefore requires a dual focus: strengthening hospital stewardship activities while also improving prescribing and dispensing practices in ambulatory care.

The observed specimen-level heterogeneity further refines the epidemiological interpretation. Urinary isolates of *K. pneumoniae* and *P. aeruginosa* demonstrated significantly higher MDR prevalence than bloodstream or mucosal isolates. This may be consistent with several possible explanations, including differential antimicrobial exposure, infection complexity, or clinical testing patterns associated with urinary tract infections; however, causal mechanisms cannot be determined from the available surveillance data. In contrast, *E. coli* showed no statistically significant variation in MDR across specimen types, suggesting broader dissemination of resistance traits across clinical syndromes. Combined with this, there is a need for rapid diagnostic testing expansion, risk-stratified empiric algorithms, and de-escalation protocols once susceptibility results are available. INAG explicitly states that antibiotic therapy should be reviewed continuously for adjustment or discontinuation [33]. Embedding structured 48‐72-hour review checkpoints into routine clinic practice, especially in hospitals, may mitigate unnecessary prolonged broad-spectrum exposure. The high MRSA prevalence also suggests a need to review empiric coverage for suspected staphylococcal infections, particularly in hospitalized patients. However, expanding glycopeptide or linezolid use without stewardship oversight would be counterproductive.

### Policy and Surveillance Implications

The modest number of isolates relative to population catchment size highlights structural limitations in routine AMR surveillance in Iraq. Because testing was based on routine clinician-directed diagnostic practice rather than systematic sampling, the dataset may be enriched for clinically complex, severe, treatment-refractory, or health care-exposed cases. This potential selection bias should be considered when interpreting resistance frequencies and limits direct extrapolation to population-level prevalence or nationally representative pathogen distributions. Current surveillance remains largely clinician-driven and laboratory-based, with limited denominator integration or standardized sampling. Strengthening surveillance will require integrated laboratory information systems, standardized reporting frameworks, improved diagnostic capacity, and denominator-informed metrics linked to health care usage. More systematic diagnostic sampling across common infection syndromes could generate surveillance data that are less biased toward resistant phenotypes and more representative of routine clinical microbiology, thereby improving empiric therapy guidance and preservation of effective Access antibiotics.

The Iraqi National Action Plan on AMR (2018‐2022) emphasized strengthening surveillance and rational prescribing [9]. While this study contributes multicenter evidence, it also highlights persistent resistance challenges and the need for unified laboratory reporting aligned with WHO GLASS standards [21]. Although GLASS provides essential surveillance infrastructure, sentinel reporting may underrepresent subnational variability [6]. Regular antibiogram reporting, interoperable data systems, and coordinated AWaRe-focused education and quality indicators could support more evidence-based prescribing and progress toward UN General Assembly AMR targets [48].

### Limitations

This multicenter analysis incorporated deduplicated microbiological data from four Iraqi governorates using internationally recognized GLASS-aligned methodological principles and standardized multidrug resistance definitions, improving comparability with regional and global surveillance reports [21,24]. However, several limitations warrant careful consideration. First, the study included selected governorates representing approximately 10% of the Iraqi population and should not be interpreted as nationally representative. Data were generated through routine clinician-directed microbiological testing without systematic sampling frameworks or denominator information. Consequently, the dataset may be biased toward patients with more severe illness, treatment failure, health care exposure, or increased clinical suspicion of resistant infection. These factors may influence observed resistance proportions and constrain generalizability beyond participating laboratory settings. The study did not have access to denominator data on total hospital admissions, outpatient consultations, infection episodes, or total specimens processed; therefore, resistance proportions cannot be interpreted as population incidence or prevalence estimates. Although the study included geographically distinct governorates and major referral laboratories, the sampling strategy was not designed to produce nationally representative AMR estimates. Findings therefore reflect resistance patterns within selected public-sector referral settings rather than the entire Iraqi health care system. Resistance estimates may differ in non-participating governorates, private-sector laboratories, smaller health care facilities, or rural settings with different diagnostic access and prescribing practices. The study categorized isolates by inpatient versus outpatient laboratory setting; however, outpatient status should not be interpreted as equivalent to community-acquired infection. The dataset did not include information on prior hospitalization, health care contact, referral status, invasive procedures, or recent antibiotic exposure. Consequently, comparisons between inpatient and outpatient isolates reflect clinical care setting rather than definitive health care-associated versus community-acquired infection epidemiology. Second, data were derived from the principal public tertiary hospital laboratory in each city. While these centers function as major referral facilities, they do not capture all microbiological testing conducted in smaller hospitals, private laboratories, or rural health facilities. Consequently, the findings may not fully represent resistance patterns across the entire health care system. Third, culture and susceptibility testing was ordered at clinician discretion. Although testing is provided free of charge in the included public hospitals, it is plausible that testing was more frequently performed in patients with severe disease, hospitalization, treatment failure, or diagnostic uncertainty. Such selective testing may enrich the dataset for more resistant organisms and potentially overestimate resistance prevalence relative to all infections occurring in the community. This limitation is inherent to many laboratory-based AMR surveillance systems internationally [21,29]. Laboratory testing panels were not completely uniform across centers, resulting in variable denominators for some antibiotic classes. Phenotypic confirmatory testing and molecular characterization of ESBL production were not performed. Accordingly, third-generation cephalosporin resistance findings should be interpreted as proxy indicators of probable ESBL activity rather than definitive measures of confirmed ESBL prevalence. As a cross-sectional laboratory-based analysis, causal inferences regarding drivers of resistance cannot be drawn. Although subgroup comparisons were undertaken by clinical setting, specimen type, and governorate, the dataset lacked several critical patient- and system-level variables necessary for robust multivariable adjustment, such as prior antimicrobial exposure and recent hospitalization. Consequently, observed differences should be interpreted as descriptive surveillance findings rather than adjusted estimates of independent resistance determinants. Future studies using prospectively collected clinical datasets or linked health system data would be valuable to enable more robust multivariable analyses of resistance risk factors. Furthermore, the analytical approach was intentionally descriptive and surveillance-oriented. Although subgroup comparisons by clinical setting and specimen type were undertaken to characterize variation in resistance patterns, the study did not use surveillance-oriented or adjustment for multiple comparisons because these analyses were outside the primary objectives of this multicenter surveillance synthesis. Accordingly, subgroup findings should be interpreted as descriptive rather than adjusted inferential estimates.

### Conclusions

This multicenter surveillance analysis highlights substantial multidrug resistance and clinically important resistance among priority bacterial pathogens across four Iraqi governorates. Beyond the observed microbiological burden, the findings illustrate important structural gaps in routine AMR surveillance, including limited denominator integration, heterogeneous laboratory reporting, and dependence on clinician-directed testing practices. Strengthening integrated laboratory information systems, standardized antibiogram reporting, and linkage between surveillance outputs and antimicrobial guideline revision processes will be essential for improving stewardship effectiveness and informing evidence-based AMR policy in Iraq. More broadly, the study demonstrates the feasibility and value of multicenter laboratory data aggregation in fragile and resource-constrained settings, providing potentially transferable lessons for AMR surveillance strengthening in other LMIC and postconflict health systems. While findings are not intended to provide population-level national estimates, they offer operationally relevant surveillance evidence to inform laboratory strengthening, stewardship planning, and future development of integrated AMR monitoring systems in Iraq.

## Supplementary material

10.2196/94436 Checklist 1 STROBE checklist.

## Abbreviations

AMR: antimicrobial resistance
AMS: antimicrobial stewardship
AST: antimicrobial susceptibility testing
AWaRe: Access Watch Reserve
ESBL: extended-spectrum beta-lactamase
GLASS: Global antimicrobial resistance and use surveillance system
LMIC: low-and middle-income countries
MDR: multidrug-resistant
MRSA: methicillin-resistant Staphylococcus aureus
WHO: World Health Organization
