# Supplementary material for: Antimicrobial Resistance Across Four Iraqi Governorates: A Multicenter Cross-Sectional Surveillance Analysis With Implications for Antimicrobial Stewardship Policy and WHO AWaRe–Aligned Public Health Strategies
Source: JMIR Public Health Surveill. 2026 Aug 20;12:e94436. doi: 10.2196/94436 (PMC13492190; doi:10.2196/94436)
Supplement: Supplementary file 1 [file publichealth-v12-e94436-s001.pdf]

STROBE Checklist for Cross-Sectional Studies

Manuscript Title: Multicentre Antimicrobial Resistance Surveillance Across Four Iraqi Governorates: Implications for Stewardship Policy and WHO AWaRe–Aligned Public Health Strategies

Journal: JMIR Public Health and Surveillance

Reporting Guideline: STROBE Statement – Checklist of items that should be included in reports of cross-sectional studies

Section/Item	Item No.	Recommendation	Reported on Page(s) / Section(s)
**Title and Abstract**			
Title and abstract	1(a)	Indicate the study's design with a commonly used term in the title or the abstract	Title; Abstract Methods
	1(b)	Provide in the abstract an informative and balanced summary of what was done and what was found	Abstract
**Introduction**			
Background/rationale	2	Explain the scientific background and rationale for the investigation being reported	Introduction
Objectives	3	State specific objectives, including any prespecified hypotheses	Final paragraph of Introduction
**Methods**			
Study design	4	Present key elements of study design early in the paper	Methods: Study design and setting
Setting	5	Describe the setting, locations, and relevant dates, including periods of recruitment, exposure, follow-up, and data collection	Methods: Study design and setting
Participants	6(a)	Give the eligibility criteria, and the sources and methods of selection of participants	Methods: Study population and eligibility criteria
Variables	7	Clearly define all outcomes, exposures,	Methods: Study population and eligibility

Section/Item	Item No.	Recommendation	Reported on Page(s) / Section(s)
		predictors, potential confounders, and effect modifiers	criteria; Antimicrobial susceptibility testing
Data sources/measurement	8*	For each variable of interest, give sources of data and details of methods of assessment (measurement)	Methods: Specimen collection and microbiological identification; Antimicrobial susceptibility testing
Bias	9	Describe any efforts to address potential sources of bias	Methods: Study population and eligibility criteria; Discussion: Limitations
Study size	10	Explain how the study size was arrived at	Methods: Study design and setting
Quantitative variables	11	Explain how quantitative variables were handled in the analyses	Methods: Statistical analysis
Statistical methods	12(a)	Describe all statistical methods, including those used to control for confounding	Methods: Statistical analysis
	12(b)	Describe any methods used to examine subgroups and interactions	Methods: Statistical analysis
	12(c)	Explain how missing data were addressed	Methods: Study population and eligibility criteria
	12(d)	If applicable, describe analytical methods taking account of sampling strategy	Methods: Study design and setting
	12(e)	Describe any sensitivity analyses	Not applicable
**Results**			
Participants	13(a)	Report numbers of individuals at each stage of study	Results: Study population and specimen characteristics
	13(b)	Give reasons for non-participation at each stage	Not applicable
	13(c)	Consider use of a flow diagram	Not applicable
Descriptive data	14(a)	Give characteristics of study participants and information on	Table 1; Results: Study population and

Section/Item	Item No.	Recommendation	Reported on Page(s) / Section(s)
		exposures and potential confounders	specimen characteristics
	14(b)	Indicate number of participants with missing data for each variable of interest	Methods: Study population and eligibility criteria
Outcome data	15	Report numbers of outcome events or summary measures	Results; Figures 1–4
Main results	16(a)	Give unadjusted estimates and, if applicable, confounder-adjusted estimates and their precision	Results: Composite resistance indicators and multidrug resistance
	16(b)	Report category boundaries when continuous variables were categorized	Table 1
	16(c)	If relevant, consider translating estimates of relative risk into absolute risk	Not applicable
Other analyses	17	Report other analyses done, such as subgroup analyses and interactions	Results: Specimen-specific MDR variation
**Discussion**			
Key results	18	Summarise key results with reference to study objectives	Discussion: Key findings
Limitations	19	Discuss limitations of the study, taking into account sources of potential bias or imprecision	Discussion: Limitations
Interpretation	20	Give a cautious overall interpretation of results considering objectives, limitations, multiplicity of analyses, results from similar studies, and other relevant evidence	Discussion
Generalisability	21	Discuss the generalisability (external validity) of the study results	Discussion: Policy and surveillance implications; Conclusions
**Other Information**			

Section/Item	Item No.	Recommendation	Reported on Page(s) / Section(s)
Funding	22	Give the source of funding and the role of the funders	Funding statement
Ethical approval	—	Provide information on ethical approval and consent procedures	Methods: Ethical considerations
Data availability	—	Describe data sharing and availability arrangements	Data Availability statement

*Give information separately for exposed and unexposed groups if applicable.

Reference

von Elm E, Altman DG, Egger M, et al. The Strengthening the Reporting of Observational Studies in Epidemiology (STROBE) statement: guidelines for reporting observational studies. *Lancet.* 2007;370(9596):1453-1457.
